# Exploring Identity in Muslim Moroccan and Pakistani Immigrant Women

**DOI:** 10.5964/ejop.v11i1.844

**Published:** 2015-02-27

**Authors:** Cristina Giuliani, Semira Tagliabue

**Affiliations:** a Department of Psychology, Università Cattolica del Sacro Cuore, Milano, Italy; b Department of Psychology, Università Cattolica del Sacro Cuore, Brescia, Italy; Aalborg University, Aalborg, Denmark

**Keywords:** cultural identity, Pakistani and Moroccan immigration, Muslim women, qualitative analysis

## Abstract

This study presents a qualitative investigation of how Muslim Moroccan and Pakistani female immigrants living in Italy conceptualize their cultural identity. Ten Moroccan and 10 Pakistani (adolescent and adult) women were interviewed through in-depth semi-structured interviews. The interviewees expressed a strong attachment to their culture of origin: their religion is a crucial aspect of their identity, along with certain cultural rules and traditional values. At the same time, both Moroccan and Pakistani participants were ambivalent toward and experienced difficulties in developing a connection to the host country, although the two groups exhibit their lack of connection to their host country in different ways: Moroccans’ self-representation is marked by a sense of foreignness and by a lack of an emotional connection with places where they are living while Pakistanis tend to express cultural distance and conflict with the host culture’s values. For both the Moroccan and Pakistani groups, the challenge of integration and biculturalism seems demanding in the Italian context and is marked by a deep feeling of emptiness, a lack of an emotional bond with the new country, and a strong cultural ambivalence. Finally, narrative themes are articulated across four interrelated dimensions (cultural, religious, gendered, spatial), revealing interesting differences based on national origin and generation.

## Introduction

In Europe, as in countries with a longer history of immigration such as the United States, Canada, Australia, and New Zealand, Muslims constitute a growing new immigrant population from a wide range of African and Middle Eastern countries. An expanding body of empirical work has focused on the acculturation process of Muslim immigrants living in Western countries, in particular in the post-9/11 era (Sirin & Fine, 2007). The term *acculturation* has been used to refer to the “process of cultural and psychological change that takes place as a result of contact between two or more cultural groups” (Berry, 2006, p. 13), leading to some long-term psychological and social adaptations of an individual or group. In this process, cultural identity, considered as a multidimensional construct linked to a sense of self as a member of one or more groups (e.g., Berry, Phinney, Sam, & Vedder, 2006), has been frequently analysed both in young and adult immigrants. Several authors have pointed out that the negotiation and interplay among religious, cultural, ethnic, and national dimensions of identity represent a complex challenge, one that is generally considered more demanding for second-generation children of immigrants than for the first-generation immigrants (Berry et al., 2006; Britto & Amer, 2007; Phinney, 1990; Stuart, Ward, & Adam, 2010). Children of immigrants struggle with combining loyalty with cultural and religious heritage and host society values earlier in their developmental and socialization process than do their adult counterparts.

Difficulties related to the acculturation experience have been documented less in North American and Australian studies than in European. North-American and Australian studies have found that adolescent and young adult children of Muslim immigrants tend to choose an integrated or bicultural identity (Britto & Amer, 2007; Stuart et al., 2010), and that female adolescents are able to navigate more fluidly than their male peers between dual cultural frameworks, without experiencing a high level of ambivalence or conflict, and without feeling “split” between cultures (Sirin, Bikmen, et al., 2008; Sirin & Fine, 2007, 2008; Zaal, Salah, & Fine 2007). Moreover, it seems that a strong Muslim identification is balanced by participation in the broader society (Sirin, Bikmen, et al., 2008; Stuart et al., 2010). In Europe, however, Muslim immigrant youth rarely achieve an integrated or bicultural identity: they seem to prefer an ethnic- over a national identification (Berry et al., 2006; Crul & Doomernik, 2003; Heim, Hunter, & Jones, 2011; Stevens, Pels, Vollebergh, & Crijnen, 2004; Vedder, Sam, & Liebkind, 2007). Moreover, in contrast to immigrant Muslim boys, immigrant Muslim girls show an ambivalent acculturation pattern (Stevens et al., 2004, p. 699).

### Characteristics of the European Context of Immigration

Some distinctive characteristics of the European context of immigration need to be considered in order to better understand the problematic acculturation patterns for young immigrant Muslims in European countries (Alba, 2005; Duyvendak, 2011; Leyendecker, 2011). First, the close geographic proximity between countries facilitates the contact with friends left behind and with one’s own culture of origin. Inexpensive flights, the possibility of spending yearly holidays in the homeland, and new technologies allow people to stay closely connected to friends and family in the country of origin. Second, the high level of negative biases, intolerance toward low-status immigrants, religious – institutional- and societal discrimination, and the poor school and professional integration experienced by Muslim immigrants in many EU countries all represent both a barrier against cultural integration and identification with the dominant group (Adida, Laitin, & Valfort, 2010; Allen & Nielsen, 2002; Ambrosini, 2013; Berry et al., 2006). Lastly, European-specific migration patterns, which today rely on a legal system of family reunification and formation, do not allow for the emergence of a true third generation of immigrants, that is, a generation of offspring with both parents born in the host country. Since the 1990s, being brought as a spouse to the destination country has been - and still is - one of the principal legal means of gaining entry into Europe for new immigrants (de Haas, 2007; Leyendecker, 2011). Indeed, family and parents often prefer their children to marry a partner from the country of origin. Furthermore, these marital choices are often strengthened by strong cultural norms regarding consanguineous marriages and family ties (Bertolani, Rinaldini, & Bordogna, 2014; Erricchiello, 2011; Reniers, 2001). In Europe, unlike in the United States, third- and fourth-generation immigrants frequently live in families in which parents have a “mixed generational status” (Leyendecker, 2011, p. 5): for instance, one parent may be born or have grown up in Europe, while the other in the country of origin, carrying with him or her his own culture and language. Thus, the combined effects of (1) geographical proximity and close connection to the country of origin, (2) intolerance and discrimination toward Muslims, and (3) European-specific migration patterns explain young Muslims’ struggles to integrate feelings of belonging to both ethnic and host cultures.

### Characteristics of the European Research About Muslims

Comparing European studies with North-American and Australian research is difficult for yet another reason, in this case related to sampling. North American and Australian studies tend not to distinguish among different ethnicities; they might, for example, investigate the acculturation experience of Muslim immigrants or Arab Americans, regardless of their specific geographical provenience. In contrast, European studies, although scarce, have tended to focus on one specific immigrant ethnic group: in general, the largest and most-established in the country where the research is performed. For example, Spanish, French, and Dutch studies have focused specifically on Moroccan immigrants (García-Sánchez, 2010; Sabatier, 2008; Stevens et al., 2004; Stevens, Vollebergh, Pels, & Crijnen, 2007a, 2007b); British, Finnish, and Norwegian studies on Pakistani immigrants (Campbell & McLean, 2003; Oppedal & Røysamb, 2007); and studies from the north western area of Europe (Germany, Finland, Norway, and The Netherlands) on Turkish immigrants (Crul & Doomernik, 2003; Oppedal & Røysamb, 2007; Vedder et al., 2007). Yet few published studies have examined immigrant patterns of acculturation in those European Mediterranean countries —in particular Spain and Italy — which only emerged as new destination countries for immigrants beginning in the 1980s. Research in the field is particularly lacking in Italy (Salih, 2000, 2009), with a few exceptions (Regalia & Giuliani, 2012), which found that Muslim immigrants to Italy collectively experienced a fragile and complex transition experience, characterized by deep feelings of solitude and social isolation, high levels of perceived discrimination, and strong religious identification.

### Moroccan and Pakistani Communities in Italy

In Italy today, Muslim Arab, North African, and South Asian immigrants constitute approximately 20% of the legalized immigrant population (Blangiardo, 2013; Istituto italiano di statistica [ISTAT], 2014). Within those immigrant populations, Moroccans and Pakistanis comprise, respectively, the largest non-E.U. immigrant populations residing in Italy (with 524,775 Moroccans officially residing in Italy on January 1, 2014), and one of the groups that has experienced the highest increase during the last decade (with 106,485 Pakistanis officially residing in Italy on January 1, 2014) (ISTAT, 2014).

Despite their similarities as regards their numbers, these two groups have differing migratory histories and characteristics. Migration from the country of Morocco began in the mid-1980s, whereas migration from Pakistan started in the second half of the 1990s. For both nationalities, the first migration wave was male-dominated, though this trend was gradually counterbalanced by the arrival of women and children within the scope of family reunification (Ambrosini, 2013; ISTAT, 2014).

The immigrant experience is related to the political and social-economic conditions in the immigrant’s country of origin: Morocco is considered to be a more developed and dynamic country with a Gross Domestic Product (GDP) per capita of US$3082 (WHO, 2012), while Pakistan is a poorer country with a GDP per capita of US$1183 (WHO, 2012). Both are Muslim societies, but, whereas reformist and modernized ideas about women’s rights have spread throughout Moroccan society, in Pakistan the fundamentalist Islamic tradition is still strong (Giunchi, 2012).

Most Moroccan and Pakistani immigrants in Italy are living in the northern part of the country: the former settled predominantly in urban areas, while the latter settled in rural or suburban areas (Blangiardo, 2013). These immigrants perform principally lower-skilled work (the building trade, domestic work, agriculture, small industries) (Ambrosini, 2013). The percentage of female Moroccan immigrants in Italy (43,9%) today almost equals that of male immigrants (56,1%) from the same country. In contrast, in the Pakistani community the percentage is lower (33% versus 67%) (ISTAT, 2014). Moreover, the levels of participation of women in the workforce are different for Moroccan and Pakistani communities: while 22% of Moroccan women are part of the Italian labor market, the same is true for only 3% of Pakistani women (Centro Studi e Ricerche [IDOS], 2014).

Comparisons between Moroccan and Pakistani immigrants are interesting for socio-demographic, historical, political, religious, and cultural reasons (de Haas, 2007; Giunchi, 2012; Zaman, Stewart, & Zaman, 2006). Moreover, the exploration of how Moroccan and Pakistani immigrants describe their experience of migration, and identification of similarities, and differences between those two groups and between first and second generation immigrant women is essential, as, currently, the available literature contributes little (Oppedal & Røysamb, 2007; Vedder et al., 2007).

The aim of the current study, which concentrates on how immigrant women of two ethnic groups (Moroccan and Pakistani) and of two age groups (adult and adolescent immigrants) conceptualize their identity, is to fully analyse the post-migration experiences of women in correlation with their national origins and generational status.

## Method

### Participants

The 20 female immigrants who participated in the current study are a subset of the group of participants of a larger study conducted by Regalia and Giuliani (2012) from January 2008 to December 2011. Among the participants, 10 adult immigrant women (5 from Morocco, 5 from Pakistan-Punjab) and 10 immigrant girls (5 from Morocco, 5 from Pakistan-Punjab) met the following criteria and were thus selected: (1) adult immigrant women should be married, living in Italy with their family (husband and children) for at least two years; and (2) immigrant girls should be between ages 14 and 18, have immigrated to Italy through family reunification, and have lived in Italy for at least two years. All participants were selected from the northern region of Italy and identified themselves as Muslim.

At the time of the interviews, the average age of the immigrant women was 33.90 years (*SD* = 5.04; *M* = 31.60, *SD* = 6.34 for Moroccans; *M* = 36.20, *SD* = 1.92 for Pakistanis). Length of residence in Italy for women was an average of 9.60 years (*SD* = 3.57; *M* = 11.40, *SD* = 3.71 for Moroccans; *M* = 7.80, *SD* = 2.59 for Pakistanis). All women had a low level of education (for the most part no schooling, or schooling through the first grade); Moroccan women were employed full-time or part-time in unskilled jobs, while Pakistani women were homemakers.

Adolescent girls immigrated to Italy when they were, on average, 7.80 years old (*SD* = 5.05; *M* = 7.20, *SD* = 5.85 for Moroccans; *M* = 8.40, *SD* = 4.72 for Pakistanis); at the time of the interviews the average age of the adolescents was 16.70 years (*SD* = 1.57; *M* = 17.20, *SD* = 1.30 for Moroccans; *M* = 6.20; *SD* = 1.78 for Pakistanis). All adolescent girls were attending secondary level school. Average length of residence in Italy for adolescents on average was 8.40 years (*SD* = 5.25; *M* = 10.00, *DS* = 6.40 for Moroccans; *M* = 6.80, *DS* = 3.83 for Pakistanis).

All participants were fluent in Italian, and all interviews were conducted in Italian.

### Materials and Procedure

After obtaining written individual consent (and parental consent for adolescent girls) to participate under the condition of anonymity, participants were asked to answer a brief demographic questionnaire, which gathered relevant background information, and to complete an in-depth, semi-structured individual interview conducted by two expert interviewers. Approval from the Ethical Committee of the Psychological Department Catholic University of Milan was sought and received.

Specific areas of inquiry in the semi-structured interviews included personal and family immigration history (e.g., “Tell me about your arrival in Italy”; “Can you describe your experience?”), life experience in the host country (family, school, work, leisure), self-representation (e.g., “Could you talk about yourself through images, things that are meaningful for you?”), comparisons between homeland and host country, social context and social relationship, future projects, and expectations about the future.

The interviews took place in the respondents’ homes or in public spaces (i.e., library, school), and respondents were offered a 10-euro gift certificate to a local market for their participation.

### Coding

The interview transcripts were analysed with grounded theory methodology, using open and axial coding (Strauss & Corbin, 1990). ATLAS.ti Scientific Software Development (2013) software was employed throughout the qualitative data analysis process to code the primary documents. A team of two researchers independently analysed the verbal data following completion of the systematic steps. First, they openly coded the data, inductively developing a preliminary list of *codes*, describing the content of selected narrative quotes. The term *codes* refers to the initial labels used to characterize the transcript quotations^i^. Next, the researchers grouped the codes into higher-level conceptual categories, called *themes.* Finally, the themes were grouped into more comprehensive conceptual categories which indicate the conceptual macro area of interest.

Themes and main thematic dimensions were then analysed further through clustered matrices, which allowed the subject to describe narratives, but taking into account country of origin (Morocco/Pakistan) and generation (adult/adolescent). For purposes of publication, the quotations have been translated from Italian to English by the authors.

## Results

### Themes and Dimensions Identified

Collected data disclosed rich qualitative information concerning the way in which immigrant women and girls describe their life experiences and their cultural belonging. Several themes emerged from these narratives, which are described here in depth. Using these themes as a starting point, four overarching and interrelated thematic dimensions were identified: *Cultural dimension of identity* was used to indicate times when participants described themselves in relation to cultural systems, revealing cultural orientations, behavioural preferences, and values concerning both their heritage group and the larger society in which they now live. The *Gender dimension of identity* was employed for times when participants described their experiences as women (values, behaviours, beliefs). The *Religious dimension of identity* referred to those times when participants valued their religious affiliation and described their thoughts and engagement in devout practices*; Spatial dimension of identity* indicated times when participants expressed their attachment to physical places, the emotional bond with a geographical region or land, the strength of their feeling at home, or, conversely, the lack of this feeling.

As shown in Table 1, each of these four thematic dimensions referred to specific themes.

**Table 1:** Themes and Thematic dimensions of identity

Theme	Thematic Dimension
Culture of Origin Caught between two cultures Apart from host culture Italian behavioural assimilation Culture of host country	Cultural dimension of identity (*n* = 97)^a^
Independent and free woman Traditional woman	Gender dimension of identity (*n* = 55)
Muslim affiliation Religious practices	Religious dimension identity (*n* = 54)
Without a land Motherland bond Host country bond	Spatial dimension of identity (*n* = 51)

^a^Numbers reported refer to the total quotes for each main theme identified.

#### Cultural Dimension of Identity

Most of the interviewees’ self-narratives developed around the main theme *cultural dimension of identity,* and, within this dimension, immigrant women and girls valued their relationship with their *culture of origin.* In a few cases, they expressed attachment and openness to the *culture of the host country.* These participants were very focused on traditional values, rules, and habits. For instance, immigrants stressed solidarity among families, family support, loyalty and respect toward one’s culture of origin and a collectivistic orientation, as indicated by statements like the following:

In our culture we help each other; there, I would find the warmth, the affection among people, but here people are too busy and don’t have time to devote to others, sometimes even to their own family (Participant 5, Moroccan woman, 34 years old).

Participants often referred to cultural rules:

Our culture is a culture inherited from India….Although we are Muslims, our traditions date back to India (Participant 16, Pakistani adolescent, 18 years old).

Even if I live here, I live the way I lived in Morocco. My life has not changed because I cannot go out in the evening; we have rules. It is the same culture, nothing different from before (Participant 11, Moroccan adolescent, 18 years old).

My husband and I have explained the rules of our culture to her. Our task is to explain what is right and what is wrong in our culture (Participant 6, Pakistani woman, 33 years old).

Participants referred to their traditional habits about maintaining their mother tongue, as well as preferences in food and television programs:

Of course, my parents try to encourage us to speak Arabic at home; after all, outside the home we speak only Italian, so inside the home they say “Speak Arabic, speak Arabic”, in order to preserve our own language (Participant 14, Moroccan adolescent, 18 years old).

I speak Urdu, … Punjabi. It is my native language (Participant 17, Pakistani adolescent, 15 years old).

We don't speak Italian at home, only Arabic. We are still native … we have not changed much here (Participant 5, Moroccan woman, 34 years old).

I do not watch Italian TV, I watch only Pakistan TV…I like the programs. There are many TV shows where I can see weddings… I watch those shows…because we have a Pakistani and an Indian channel (Participant 19, Pakistani adolescent, 16 years old).

Those who felt a strong connection to their culture of origin also felt a weak link with the Italian host culture, which they perceived as a distant culture that conflicted with their own culture. In line with this, the acculturative experience was characterized by struggle and difficulties, expressed as the feeling of being caught between two cultures:

My thoughts are confused, so my words are confusing (Participant 20, Pakistani adolescent, aged 18 years).

The difficulty that I have found is in comparing the Italian and Pakistani cultures; because what I find normal in Pakistani culture, I do not find normal here…I am not able to fairly compare them, I don’t know how to do it. That is, many times teachers say to me: "Come on, do it this way." Seen through the lens of an Italian this is not such a bad thing ...but through the lens of a Pakistani, it is not acceptable. I would try to avoid that thing (Participant 16, Pakistani adolescent, 18 years old).

I live split in half, 50% Italian, 50% Moroccan (Participant 14, Moroccan adolescent, 18 years old).

I must become a part of this culture, which is bit different; I cannot put the two things together… “I must become a part of this culture”: I’ve been telling myself this for the last eight months (Participant 17, Pakistani adolescent, 15 years old).

or of being apart from Italian culture:

There are some things here that really make me feel homesick for my country, because they make me feel different: not suitable for this place, not suitable for this country (Participant 15, Moroccan adolescent, 17 years old).

And the bad thing is that I do not feel comfortable with Italians. That is, they are different, we are different (Participant 11, Moroccan adolescent, 18 years old).

I did not know all the people here were different from us ...that the mentality of an Italian is different from that of a Pakistani, even though I’ve been here from 10 years. I'm half Italian, but in any case the way of thinking is different, so it would be easier to stay with a person with a similar mentality to yours …But I think that the mind-set is different: I cannot reason with the Italian people (Participant 16, Pakistani adolescent, 18 years old).

Moreover, disorientation, confusion and stress seemed to characterize the challenge of the intercultural dialogue.

Positive orientation toward Italian culture was confined to efforts of behavioural assimilation concerning more peripheral domains of acculturation, namely those in material and instrumental areas:

Christmas…in truth we who are Muslims should not celebrate it. We celebrate it, but in a different way, whereas for you it is more symbolic: you pray, we only exchange gifts (Participant, 12, Moroccan adolescent, 15 years old).

It clear that I am Italian, especially from my behaviour. And, okay I behave like an Italian; I do everything they do (Participant 12, Moroccan adolescent, 15 years old).

I am like the Italians in my gestures, because I make so many gestures. I gesticulate, yes, and in Pakistan in my family nobody gesticulates, they don't make all these gestures! (Participant 16, Pakistani adolescent, 18 years old).

Thus, these participants felt Italian when making a gesture, eating pizza, or exchanging presents on Christmas day. Only rarely did the interviewees reveal a deep and meaningful identification with the basic elements of mainstream culture, with its symbolic and ideological elements, such as ways of thinking, principles, beliefs, and values. Therefore it was unusual to find a sentence such as the following in which the participant expressed a strong connection to the Italian culture:

[The women] of my country are different from me; you cannot communicate with them. I prefer to interact with Italian women, rather than with people from my country because we [Italians and I] think the same way, about so many things (Participant 4, Moroccan woman, 23 years old).

#### Gender Dimension of Identity

Gender self-representation seemed to give equal value to, on the one hand, the image of a free woman who is emotionally independent from the family and adamant about her choices, and, on the other, the image of a traditional woman who is loyal to cultural gender norms, is located within the physical space of home, is governed by family members in her social relationships, and modest and decorous in her dress and everyday behaviour. This result clearly exemplified the cultural gap and the struggles that the participants live with during their contact with Western culture. Indeed, interviewees spoke about their gender identity in various ways. Examples of quotes in which the participants described themselves as free and independent women, include the following:

I can dress the way I want - well, perhaps not in a loincloth (Participant 12, Moroccan adolescent, 15 years old).

At least inside house, with my husband, I have the freedom to state my opinion. I am involved in the raising of my children. … in Morocco, the man has the power. Here, instead, I can say to him: "We can do this, we can do that…. I can allow my daughter to dress as she wants. If she wants to pray, she prays. She is free to do what she feels like doing In Morocco no, this thing cannot be done”. (Participant 1, Moroccan woman, 38 years old)

In my country the man has more influence because he is strong, but this is not acceptable for me, I want to change this, because it is not all right, always the men….Also the women must do something…Now I have more chances to express myself, I am free and independent, to study, to achieve a job and professional carrier. (Participant 17, Pakistani adolescent, 15 years old)

There, you must not have a boyfriend, that is, only when you get married, you can see him, only when you get married, you can date….There they have the laws, therefore you cannot have a boyfriend, that is, it is not a law, however the families that are in this way, you cannot engage with the person that you want, those things. In Italy, you are more free. Here, I can be engaged to whomever I want. (Participant 12, Moroccan adolescent, 15 years old)

In Italy you are a little, how to say, more free…that is, also in Pakistan you are a little free, however it is in a different way. There, you must also follow the religion a bit, instead here you do whatever you want. (Participant 14, Pakistani adolescent, 14 years old)

At the same time, there were many participants who focused on their traditions:

I do what my husband tells me to do. (Participant 9, Pakistani woman, 38 years old)

Women have too much freedom, and I do not like it. Thus, not only girls, but also women…Because here they decide what they want to do, they do not think to the future, as if someone discuss with her husband, they have to separate…This is what I see, then I do not know. Instead, in our culture, we really think to go on together, husband and wife, until the end. (Participant 10, Pakistani woman, aged 37 years)

I do not like the coed school, boys and girls together. (Participant 19, Pakistani adolescent, 16 years old)

I am modest, reserved with men together (Participant 17, Pakistani adolescent, 15 years old)

I do not like to spend time with boys. (Participant 18, Pakistani adolescent, aged 14 years).

I always wear something to cover me up in front…. then I put the short T-shirt….No, I meant to say, long T-shirt, because yes, you know, to cover you. (Participant 20, Pakistani adolescent, 18 years old)

#### Religious Dimension of Identity

More coherent representation was found for the theme of *religious dimension of identity*: Muslim affiliation and personal engagement in devout practices represented fundamental aspects of the immigrants’ identity. In the self-description, participants cited several aspects linked to the Muslim faith, such as their choices regarding clothes or prayers, illustrating that their affiliation with the Islamic religion was an important aspect of their identity.

Everyone keep religion as an important thing, how we are Muslim. (Participant 4, Moroccan woman, 23 years old)

I am Muslim, not Christian. (Participant 11, Moroccan adolescent, 18 years old)

My religion is Islam, I follow my religion, I believe in Allah. (Participant 19, Pakistan adolescent, 16 years old)

My life is influenced by religion, because it is a thing that, it is a bond, then here there is the Prophet. Thus, there is really the way to behave, the way….then this really is what I am. (Participant 14, Moroccan adolescent, 18 years old)

They also frequently cited Islamic beliefs and traditional religious practices concerning daily prayers, dietary practices, and celebrations.

But, well, at 2:00 p.m. when I get back from school, I have to pray again, and then again at 4:00 p.m., then again at eight o’clock. (Participant 19, Pakistan adolescent, 16 years old).

In the morning I get up and I pray…I pray for my parents. (Participant 20, Pakistan adolescent, 18 years old)

I pray, I do Ramadan, I completely follow my religion….I miss the religious celebrations, the feast at the end of Ramadan is to celebrate the end, and so the first day in which we can eat, it is a religious month in which we dedicate ourselves to the prayer, to read Koran, give importance to the religion, you should give importance to the religion all the year, but this…specifically in that month you have to care this aspect of your life…and then also the celebration for the Prophet birth.. it is important. (Participant 13, Moroccan adolescent, 18 years old)

We also pray at home. I also make the prayers every day, five times a day, so my children learn. (Participant 2, Moroccan woman, 27 years old)

#### Spatial Dimension of Identity

Finally, concerning *spatial dimensions of identity*, the most common feeling was that of being a foreigner, or an individual without a homeland.

We are foreigners, who are living in Italy (Participant 14, Moroccan adolescent, 18 years old)

Always we feel foreign, even if you have children that were born here…always we feel in a place that is not ours. You are a guest forever, you live as a guest in a home. (Participant 1, Moroccan woman, 38 years old)

You feel inside that you are not in your own country…there is no future here, I do not have to see a future here (Participant 5, Moroccan woman, 34 years old).

This negative feeling seemed mainly a description of the lack of an emotional connection to places where they are living (nation, city, neighbourhood), rather than an indication of orientation or preference in regards to cultural meanings. Italy was rarely looked upon as the motherland, and the emotional link with it was very limited; only rarely did interviewees make statements such as the following:

I feel like an Italian who was born here (Participant 13, Moroccan adolescent, 18 years old).

Indeed, the participants sometimes preferred to define themselves through the nostalgic link with their native motherland. Past experiences were evoked from the interviewees, and the coded quotes make reference to real and idealized places left behind.

I like my native country too much, my village…I feel also that it is part of my life. (Participant 16, Pakistan adolescent, 18 years old)

### Moroccan and Pakistani, Adult and Adolescent Immigrants’ Self-Representation

Interesting differences and similarities emerged when Moroccan and Pakistani narratives were compared, as well as when adult and adolescent narratives were compared.

Both adult and adolescent immigrants in the Pakistani subgroup clearly highlighted their attachment to their own culture of origin, their adherence to traditional gender image and religious affiliation, and their strong engagement in devotional practices. In contrast to those for the adult Pakistani women, the narratives for Pakistani adolescents were informed by deep feelings of disorientation and stress in intercultural contacts, feelings of being apart from the Italian people, and ambivalent and conflicting feelings regarding their gender identity. Pakistani adolescents tend to describe themselves both as traditional women in some aspects (dressing according to tradition, being modest, participating in traditional and family-arranged marriage), and as, free and independent women in others (taking opportunities to pursue life goals, continuing education, seeking professional careers and democratic rights, participating in public life, civil engagement, and freedom of speech). Thus, more so than in the adult generation, Pakistani girls expressed struggles and contradictions about the acculturative challenges they face, revealing both loyalty to the traditional gender image, and a partial valorisation of the traits of freedom, independence, and self-management possible within the new European context.

For the Moroccan subgroup, both adults and adolescents tend to describe themselves as lacking an emotional link with the geographical place they are living; they view themselves as people “without a homeland, neither here nor there,” or “foreigners in any land”. But, unlike the Pakistani subgroup, and despite this feeling of foreignness, they did not stress any attachment to their culture of origin, nor did they seem to find a strong and positive source of identification in the values and customs of their origin. That lack or weakness of identification with their culture of origin could be read in association with the absence of reports of ambivalent feelings towards the two cultures. Muslim identification seemed to provide a sort of identity marker, but unlike the Pakistani subgroup, it did not create contradictions regarding the prevalent gender image valued by them: they were clearly oriented toward valorising their image as independent and free women. Freedom for Moroccans was expressed through visible manifestations, such as non-traditional dressing, self-management in heterosexual and social relationships, and the explicit rejection of the Islamic values of the image of the woman as controlled and prudish in her behaviours.

## Discussion

The aim of this study was to explore how immigrant women conceptualize their identity by considering two different countries of origin, Morocco and Pakistan, and two different generations of female immigrants (adult and adolescent).

As found in previous explorations of the same topic, the self-description of Moroccan and Pakistani immigrants emerged as a complex association of four different and interrelated key social dimensions — cultural, gender, religious, and spatial — (Berry et al., 2006; Fried, 2000; Phinney, 1990).

Overall, and corresponding to recent international studies (Campbell & McLean, 2003; Fried, 2000; Oppedal & Røysamb, 2007; Sabatier, 2008; Stevens et al., 2004, 2007b; Vedder et al., 2007), our results present an image of immigrants who express—though in different ways — a strong attachment to their culture of origin (be it their culture of origin and their faith or their places of origin) as well as a struggle to build a link with their host country or culture.

However, immigrants talk about their experience and identity in different ways. Pakistani girls value their cultural distinctiveness, referring to their strong attachment to their culture of origin and its practices, norms, and values. For this subgroup, the culture of origin represents a positive and organized map of meaning in self-description and a key resource in interpersonal relationships (Campbell & McLean, 2003). The attachment to the culture of origin is additionally expressed by Pakistani girls through a strong Muslim affiliation, which is evidenced through their descriptions of their rigorous engagement in devout practices, and their valorisation of the traditional image of a woman. Moreover, the struggles and feelings of disorientation engendered both by the feeling of being caught between cultures and by the perception of distance from the host culture models illustrate the strength of their culture of origin. Deep conflict and ambivalence are clearly experienced by Pakistani girls with regard to gender identity as well: the highly valorised self-image as a traditional Pakistani woman is accompanied by a moderate valorisation of the traits of freedom, independence, and self-management. However, unlike adult Pakistani women, the Pakistani adolescent girls appear to be both attracted to and fearful of the Western woman’s image as free and independent.

Unlike Pakistani immigrants, Moroccan immigrants, especially Moroccan girls, frequently use spatial dimensions of identity in order to describe themselves as estranged or without a homeland (“not from around here”), pointing to the lack of a connection to the physical places in which they live. However, as they do not stress or value the attachment to their culture of origin and their religion, they do not experience strongly ambivalent feelings toward the two cultures. Like Moroccan adults, adolescent girls clearly value the image of women as being independent and free, particularly in regards to aspects related to bodily and visible manifestations of freedom (free and seductive dress, free in their romantic relationships). They refuse to label themselves according to the traditional norms of gender. The Moroccans’ limited attachment to their own culture of origin and their strong adherence to a Western model of a free woman (perhaps also stereotyped) are consistent with the pervasive sense of emotional distance experienced within their current life contexts. This distance grew out of the feeling of being without a homeland or from the nostalgic evocation of a link with a lost motherland.

In summary, Pakistanis appear strongly attached to their culture of origin and are especially loyal to a traditional image of women while, at the same time, they are simultaneously disoriented and moderately attracted to some aspects of Western culture. Meanwhile, Moroccans strongly adhere to the Western model of emancipated and independent women and they perceive a strong similarity with the Italian girls’ lifestyles. Despite this perception of similarity to their Italian counterparts, their attachment to places where they are living is limited and their self-representation is marked by a sense of foreignness. Moreover, they assign multiple and distinct meanings to freedom: when Moroccans talk about freedom, they refer to its visible manifestations, such as non-traditional dressing or self-management in heterosexual and social relationships. Instead when Pakistanis talk about freedom, they refer to opportunities to pursue educational or professional emancipation, as well as to be engaged in public and civil life.

Several factors can be hypothesized to explain the consistent differences that emerge between Pakistani and Moroccan identity representation and acculturation processes: a longer and better--established immigration history and a high level of exposure to modernized lifestyles in highly urbanized settings for the Moroccan immigrants, both in Italy and in Morocco; and the more traditional and more specific cultural heritage of the Southeast Asian community that characterizes women in the Pakistani subgroup.

In both national groups, the findings show a fragile and complex identity representation of immigrant girls and women, characterized by a pervasive sense of foreignness. This confirms the worries and the doubts about acculturation patterns of Muslims indicated by several studies that have emerged in Europe of late (Berry et al., 2006; Crul & Doomernik, 2003; Heim et al., 2011; Stevens et al., 2004; Vedder et al., 2007). However, listening to adolescent and adult immigrant women voices in the present study allows the emergence of different meanings associated with their sense of foreignness, which include not only cultural but also spatial and social components. Moroccans perceive themselves above all as individuals untied to the land where they live, but also weakly tied to their own cultural heritage. They seem to express a desire for home and belonging to a place that they seem not to have found in their current life contexts. Pakistanis, strongly attached to their heritage values and norms, express cultural distance and conflict with the host culture, which complicate the cultural negotiation process. For both the Pakistani and Moroccan groups, the challenge of integration or biculturalism seems to be demanding in the Italian context as well, and is marked by strong cultural ambivalence, a feeling of emptiness, and a lack of emotional bond with the new country.

Although the current findings are limited by the number of women interviewed and the explorative approach adopted in this study, they may help researchers to pose new questions and show the importance of not grouping Muslim immigrants into one ethnic group, but rather differentiating the study of the migration phenomenon according to both the country of origin and the country of settlement of immigrants (Berry et al., 2006; Leyendecker, 2011; Sirin, Bikmen, et al., 2008). Stronger reinforcement for this statement should come from future studies that compare the identity representation of male immigrants from different countries. It is likely that girls experience family loyalty, gender-specific cultural conflicts, and levels of acculturative stress more intensely than males, as recently documented in a number of studies (Khuwaja, Selwyn, Kapadia, McCurdy, & Khuwaja, 2007). Furthermore, a deeper consideration of the specific challenges that second generations face is needed, also differentiating early adolescence (11–14 years old) and middle adolescence (15–18 years old).

Our findings show that those aspects that define identity representation within each national group are more clearly emphasized by adolescents than by first-generation adult women. This reveals an intensification of these aspects in the self-representation of the younger generations. However, a differentiation regarding age and generational status could reveal a more detailed picture of challenges brought by the migration.

This study further supports psychosocial practitioners in their understanding of the differences of cultural identity and history, showing that interventions should be prepared according to the country of origin, and that cultural integration is a multidimensional phenomenon deserving of attention.
